# A Korean-Style Balanced Diet Has a Potential Connection with *Ruminococcaceae* Enterotype and Reduction of Metabolic Syndrome Incidence in Korean Adults

**DOI:** 10.3390/nu13020495

**Published:** 2021-02-03

**Authors:** Xuangao Wu, Tatsuya Unno, Suna Kang, Sunmin Park

**Affiliations:** 1 Obesity/Diabetes Research Center, Department of Food and Nutrition, Hoseo University, Asan 31499, Korea; niyani0@naver.com (X.W.); roypower003@naver.com (S.K.); 2 Department of Bio-Convergence System, Hoseo University, Asan 31499, Korea; 3 Faculty of Biotechnology, School of Life Sciences, SARI, Jeju National University, Jeju 63243, Korea; tatsu@jejunu.ac.kr

**Keywords:** enterotypes, dietary patterns, gut microbiota, short-chain fatty acids, inflammation, Koreans

## Abstract

Metabolic syndrome is associated with usual dietary patterns that may be involved in enterotypes. We aimed to understand the potential relationship of enterotypes and dietary patterns to influence metabolic syndrome in the Koreans. Using the Korea National Health and Nutrition Examination Survey (KNHANES)-VI in 2014, metabolic parameters were also analyzed among the dietary patterns classified by principal component analysis in Korean adults. The fecal microbiota data of 1199 Korean adults collected in 2014 were obtained from the Korea Centers for Disease Control and Prevention. Enterotypes were classified based on Dirichlet multinomial mixtures (DMM) by Mothur v.1.36. The functional abundance of fecal bacteria was analyzed using the PICRUSt2 pipeline. Korean adults were clustered into three dietary patterns including Korean-style balanced diets (KBD, 20.4%), rice-based diets (RBD, 17.2%), and Western-style diets (WSD, 62.4%) in KNHANES. The incidence of metabolic syndrome was lowered in the order of RBD, WSD, and KBD. The participants having a KBD had lower serum C-reactive protein and triglyceride concentrations than those with RBD and WSD (*p* < 0.05). Three types of fecal bacteria were classified as *Ruminococcaceae* type (ET-R, 28.7%), *Prevotella* type (ET-P, 52.2%), and *Bacteroides* type (ET-B, 42.1%; *p* < 0.05). ET-P had a higher abundance of *Prevotella copri,* while ET-R contained a higher abundance of *Alistipes*, *Akkermansia muciniphila*, *Bifidobacterium adolescentis*, and *Faecalibacterium prausnitzii*. ET-B had a higher abundance of the order *Bilophila* (*p* < 0.05). Metabolism of propanoate, starch, and sucrose in fecal microbiome was higher in ET-P and ET-R, whereas fatty acid metabolism was enhanced in ET-B. Fecal microbiota in ET-P and ET-B had higher lipopolysaccharide biosynthesis activity than that in ET-R. The metabolic results of KBD and RBD were consistent with ET-R and ET-P’s gut microbiota metabolism, respectively. In conclusion, Korean enterotypes of ET-P, ET-B, and ET-R were associated with RBD, WSD, and KBD, respectively. This study suggests a potential link between dietary patterns, metabolic syndrome, and enterotypes among Korean adults.

## 1. Introduction

Metabolic syndrome prevalence is rising worldwide, and especially in Asia, it has remarkably increased last decades due to the westernization of lifestyles, including food intake and sedentary work. Asians traditionally have an unrefined grain-based diet with vegetables for long periods, but their dietary patterns changed last decades—they consume high in refined grains, including low fiber and increasing fat intake. These dietary changes may affect gut microbiota that may interact with the host’s metabolism, and metabolic syndrome risk is modulated. Metabolic syndrome risk is related to dietary patterns and their interaction with gut microbiota, but no clear associations have been evaluated, especially in gut microbiota and dietary patterns.

Some of the essential nutrients required by the body are obtained through the production of intestinal microbes in the foods we eat. Short-chain fatty acids (SCFAs), especially acetic acid, propionic acid, and butyric acid, are partly used as an energy source in colonic epithelial cells, and they are absorbed into systemic circulation through the portal vein [1]. Studies have shown that propionate in the circulation improves insulin response and alleviates type 2 diabetic symptoms [2]. SCFAs reduce lipopolysaccharides (LPS) and tumor necrosis factor-alpha (TNF-α) that mediate inflammation [3]. In addition to the production of SCFAs, intestinal microorganisms also play a role in the vitamin B biosynthesis, metabolism of proteins and branched-chain amino acids, lipid metabolism, and other nutrient-related functions [4]. A crucial factor to affect the abundance and function of intestinal microbes is the host’s dietary intake [5]. SCFAs are mainly produced by intestinal microbes using undigested carbohydrates and proteins, including dietary fibers and gamma-polyglutamate (γ-PGA) [6]. The SCFAs production decreases, and the branched-chain amino acids production increases from intestinal microbes when the person consumes a diet with high proteins and low carbohydrates [6]. Different diet types, ages, and regions create a diverse intestinal environment in people [4,7].

A large number of intestinal microbes and their inter-individual diversity makes research in this area complicated. Therefore, classifying the diverse intestinal flora can help us simplify the results and make them better understand. In 2011, Arumugam et al. [8] proposed the concept of enterotypes, dividing fecal microbes into three enterotypes—*Bacteroides* (ET-B), *Prevotella* (ET-P), and *Ruminococcus* (ET-R)—according to the dominant bacteria present in an individual. In subsequent studies, these enterotypes observed similar or different results in different countries and populations [9]. These enterotypes have been widely accepted, and the proportion of predominant bacteria within an enterotype vary from region to region [10,11,12]. Among the various factors affecting the diversity within an enterotype, a dietary pattern is considered the most important. It has been reported that individuals consuming carbohydrate-rich diets, including dietary fiber and simple sugars, belong to the *Prevotella* enterotype, whereas those consuming protein and animal fat-rich diets are of the *Bacteroides* enterotype [13].

South Korea has a unique geographical location and distinctive culture with its specific dietary habits. In the past few decades, the Korean lifestyle and eating habits have undergone a significant change. Traditionally, rice and fermented vegetables have formed the staple diet of the Korean population. Today, their dietary patterns were changing to consume more diverse foods with consuming less rice. Food consumption was divided into those who consume a Korean-style balanced diet, as recommended by the Korean Nutrition Society, and those who prefer the Western-style diet (WSD), including high proportions of meat, noodles, bread, and fast foods [14]. Less rice consumption, even a WSD, contributes to decreasing the prevalence of metabolic syndrome in the Korean population [15]. This outcome is due to the proper proportion of carbohydrates, fats, proteins, and sufficient dietary fiber in the diets, even in a WSD. These dietary patterns may have influenced the enterotypes, and Korean enterotypes may be different from those in Western countries, even in the same enterotype, and consequently, they may affect their health benefits differently [16,17].

Studies have shown a correlation between dietary patterns and enterotypes, indicating that different dietary compositions create different gut microbiota [13]. The present study aimed to determine the relationship between metabolic syndrome and dietary patterns and the potential association between gut microbiota’s distribution and function, the enterotypes, and dietary patterns in Korean adults. Furthermore, the potential connection between the dietary patterns and the gut microbiota composition was explored in Korean adults using the semi-quantitative food frequency questionnaire of the Korea National Health and Nutrition Examination Survey (KNHANES).

## 2. Materials and Methods

### 2.1. Design and Data Collection from the Korean Population

KNHANES has been conducted annually using a rolling sampling design that involves a complex, stratified, multistage probability cluster survey of a representative sample of the non-institutionalized civilian population in South Korea [18]. KNHANES has been conducted annually with a multistage probability sample design under the Korea Centers for Disease Control and Prevention (KCDC) supervision. It evaluates the health and nutritional status of the Korean population [19]. This study utilized data obtained from the KNHANES-VI 2014 of 8944 adults (3782 men and 5162 women) aged 20–64 years old. It is a large representative population study with rigorous quality controls. The sample weights were made with sample participants to represent the Korean population, and they were used for the complex survey design, survey nonresponse, and post-stratification. KNHANES included general information (age, education level, and income level), anthropometric parameters (body weight, height, waist circumference, hip circumference, and blood pressure), biochemical parameters related to glucose and lipid metabolism and inflammation, social factors (smoking, drinking, and exercise), and dietary intake [19], and they were collected from the volunteered participants. The Institutional Review Board of the KCDC approved KNHANES (approval no. 2013-07-CON-03-4C). The study was performed under the Helsinki Declaration (of 1975, as revised in 2008).

### 2.2. Definition of Metabolic Syndrome

Based on the 2005 revised National Cholesterol Education Program Adult Treatment Panel III (NCEP ATP III) criteria for waist circumference, metabolic syndrome was defined as the presence of three or more of the following: (1) elevated blood pressure (average systolic blood pressure >130 mmHg or diastolic blood pressure >85 mmHg) or current blood pressure medication use; (2) low HDL-cholesterol level (<40 mg/dL for men and <50 mg/dL for women); (3) elevated serum triglyceride level (≥150 mg/dL) or current anti-dyslipidemic medication use; (4) elevated fasting blood glucose level (≥100 mg/dL) or current anti-diabetic medication use; and (5) abdominal obesity (waist circumference ≥102 cm for men and >90 cm for women) [19].

### 2.3. Laboratory Analysis

Blood samples were obtained in the morning following an overnight fast. The serum concentrations of glucose, high-density lipoprotein cholesterol (HDL), triglycerides, aspartate transaminase (AST), and alanine transaminase (ALT) were measured using a Hitachi automatic analyzer 7600 (Tokyo, Japan). Low-density lipoprotein cholesterol (LDL) was measured directly using a Hitachi automatic analyzer 7600. Serum C-reactive proteins were measured with an ELISA kit (Abcam, Cambridge, UK). Serum 25-OH-D levels, as an indicator of vitamin D status, were measured with a radioimmunoassay kit (DiaSorin, Stillwater, MN, USA) using a 1470 Wizard gamma counter (Perkin–Elmer, Turku, Finland). All clinical analyses were performed by the Neodin Medical Institute, a laboratory certified by the Korean Ministry of Health and Welfare.

### 2.4. Dietary Pattern Analysis

The 112 food items from the semi-quantitative food frequency questionnaire (SQFFQ) were categorized into 25 predefined food groups. These food groups were used as independent variables wherein a factor analysis was conducted to find dietary patterns using the proc factor procedure adjusted with weights. We determined the number of factors based on eigenvalues >1.5, and 3 dietary factors described the distinct dietary-pattern in the participants. The orthogonal rotation procedure (varimax) was applied in the principal component analysis (PCA), and factor-loading values ≥0.40 were considered to have dominant contributions to the distinctive dietary pattern (Table 1) [20]. Using clustering analysis (proc fastclus), the adults were divided into three cluster groups by calculating the distance from the assigned point after standardization of the data by proc stdize.

### 2.5. Collection of Fecal Microbiota Data

Data on the fecal microbiota of 1199 Korean adults aged 20–64 years old were provided from the Clinical & Omics Data Archive (CODA) department in KCDC. The fecal microbiota analysis was performed using next-generation sequencing (NGS) in the feces collected in 2014 [21]. The Institutional Review Board of the Hoseo University approved the using the CODA data (approval no. 1041231-190816-BR-094-02).

### 2.6. Fecal Microbiota Analysis

The 16S amplicon sequences were processed using Mothur v.1.36. Using the Miseq standard operating procedure (SOP), bacteria taxonomy and counts were evaluated in each fecal sample. The sequences were aligned using Silva reference alignment v.123 included in Mothur SOP (https://mothur.org/wiki/silva_reference_files (accessed on 2 February 2021)), and the taxonomy and bacteria counts of each taxonomy were determined. The relative number of bacteria was calculated in the taxonomic assignments at the family, order, and genus levels of each sample after removing operational taxonomic units (OTUs) below 10,000 reads. Principal coordinates analysis (PCoA) was conducted using the R package with the OTU-abundance table converted to relative abundance. Linear discriminant analysis effect size (LEfSe) analyses were conducted based on the genus OTU table from fecal microbiota, and this analysis was performed at http://huttenhower.sph.harvard.edu/galaxy (accessed on 2 February 2021) to explore the significant microbiota in each enterotype. Bacteria with logarithmic discriminant analysis (LDA) score >2.0 were shown as the results in each dietary pattern. The α-diversity Chao and Shannon indices were calculated using the Mothur summary.single subroutine.

The spearman correlation coefficient matrix between genus level gut microbes’ relative abundance and the 25 food groups representing dietary patterns were calculated after adjusting age, sex, and BMI using the correlation function using CODA data in the python NumPy package.

### 2.7. Gut Microbiota Metabolism Predicted by PICRUSt2 Pipeline Analysis

Using 16S rRNA gene sequences, we predicted metabolic activities of the gut microbiota using PICRUSt2. Briefly, fasta and count table files produced by Mothur were used to make a biom file. The biom file was used to predict the metabolic activities using picrust2_pipeline.py provided (https://github.com/picrust/picrust2/wiki/Full-pipeline-script (accessed on 2 February 2021)). Predicted metabolic profile for Kyoto Encyclopedia of Genes and Genomes (KEGG) Orthologues (KO) were mapped to propionate, starch and sucrose metabolism, lipopolysaccharide biosynthesis, and fatty acid metabolism using the KEGG mapper (https://www.genome.jp/kegg/tool/map_pathway1.html (accessed on 2 February 2021)) [22]. An abundance of the mapped KOs was used to draw heatmap with R using a heatmap.2 package.

### 2.8. Statistical Analysis

Statistical analyses were performed using SAS software (version 9.4; SAS Institute, Cary, NC, USA), and the KNHANES outcomes were incorporated with sample weights and adjusted analyses for the complex sample design of the survey. The results were representative of the non-institutionalized civilian Korean population. All results were expressed as means ± standard deviations.

Descriptive statistics of participants according to dietary patterns were obtained by the frequency distributions of categorical demographic variables. Their statistical significances were determined using chi-squared tests. Adjusted means of nutrient intake and biochemical parameters were calculated according to dietary patterns after adjusted with covariates, including sex, age, residence area, occupation, income, education level, marriage, drinking and smoking status, BMI, and physical activity. Statistical differences among the dietary patterns were analysis of covariance (ANCOVA) was performed, and multiple comparisons between dietary pattern groups were conducted using Tukey’s test. Adjusted odds ratio and 95% confidence intervals of metabolic syndrome and its related parameters were analyzed by logistic regression after adjusting for age, sex, BMI, smoking and drinking status, and physical activity according to dietary patterns. Statistical significance was determined at *p* < 0.05.

## 3. Results

### 3.1. Metabolic Syndrome and Its Components According to Dietary Patterns

The WSD group participants were the youngest, but those in the rice-based diets (RBD) were the oldest among all dietary groups (Table 1). Most men (74.5%) had RBD. The incidence of metabolic syndrome was lowered in the order of RBD, WSD, and Korean-style balanced diets (KBD). BMI and waist circumferences were not significantly different (Table 1). Furthermore, serum concentrations of hemoglobin A1c and triglyceride, platelet, and white blood cell (WBC) counts were higher in the RBD than the other groups, whereas systolic blood pressure (SBP), diastolic blood pressure (DBP), serum glucose, total cholesterol, HDL and LDL concentrations, serum AST and ALT activities, and glomerular filtration rate did not differ among the groups (Table 1). The participants in the KBD had low concentrations of serum C-reactive protein (CRP) and WBC count than the other groups. It suggested that the KBD had a lower inflammation status (Table 1). The participants who belonged to the KBD had the highest serum concentrations of folate, suggesting that KBD included high amounts of vegetables (Table 1). Serum V-A concentration and blood platelet were significantly higher in RBD than other groups, while serum 25-(OH)-cholecalciferol concentrations, index of vitamin D status, showed no significant difference.

**Figure 1:**
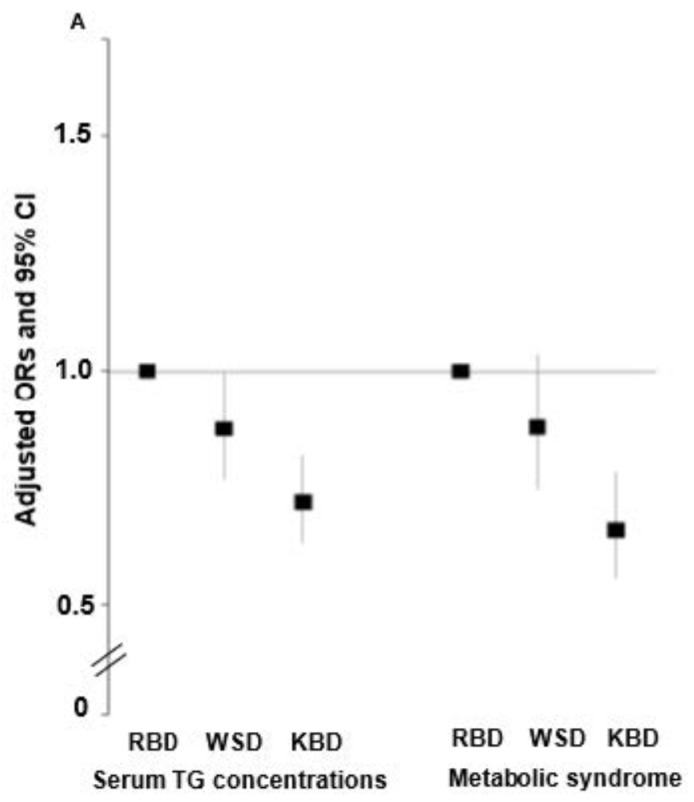

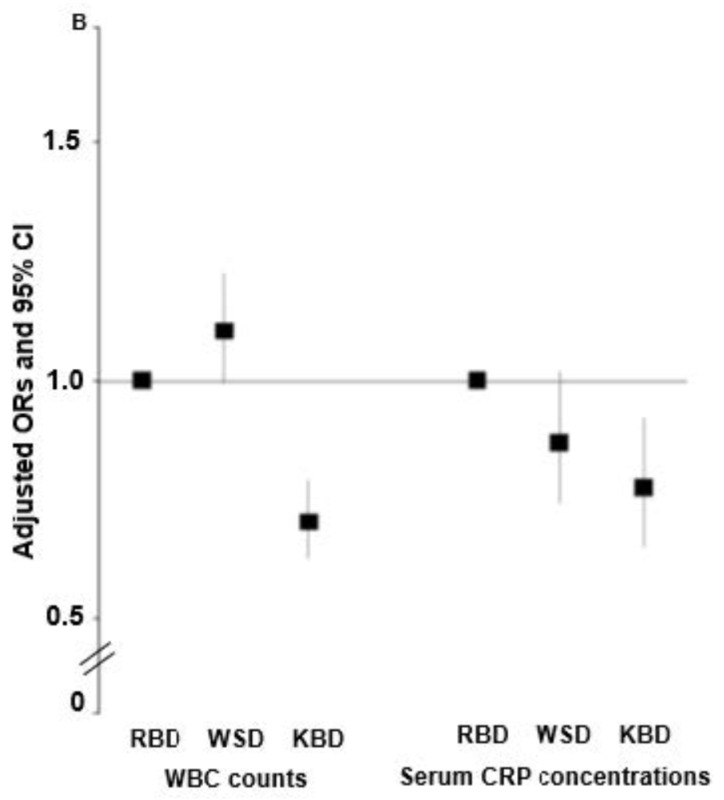
Adjusted odds ratio and 95% confidence intervals for metabolic syndrome and inflammatory indices according to dietary patterns. (**A**) Serum triglyceride concentrations and metabolic syndrome incidence. (**B**) Serum C-reactive protein (CRP) concentrations and white blood cell (WBC) counts. Adjusted covariates were age, gender, BMI, smoking and drinking status, and physical activity. The reference group was a rice-based diet (RBD). KBD, Korea balanced diet; WSD, Western-style diet.

Among the metabolic parameters, metabolic syndrome incidence had an inverse association by 0.658 times (95% CI = 0.556–0.78) with KBD compared to RBD, and serum triglyceride concentrations were inversely associated with WSD and KBD by 0.874 and 0.718 times, respectively, compared to RBD (Figure 1A). Interestingly, serum CRP concentrations and WBC counts, inflammatory indices, had an inverse association with KBD by 0.776 and 0.705 times, respectively, compared to RBD (Figure 1B). However, they were not significant associations of serum CRP concentrations and WBC counts with WSD.

### 3.2. Nutrient Intake According to Dietary Patterns

The data from KNHANES involving 8944 individuals were divided into three types of dietary patterns. Cluster 1 were consumers of a rice-based diet (RBD; *n* = 1542) and cluster 2 were mainly consumers of a Korean-style balanced diet (KBD; *n* = 1825), and cluster 3 were consumers of a Western-style diet (*n* = 5577). The KBD contained high amounts of potatoes, legumes, vegetables, mushrooms, fruits, fish, seaweeds, kimchi, and pickles. The WSD included animal foods, such as noodles, bread, meat, fast foods, mixed dishes, and sugar drinks. The RBD was characterized by refined rice, alcohol, and caffeinated drinks (Table 2). Energy intake was higher in KBD than in WSD and RBD (*p* < 0.05). The carbohydrate intake (energy %) was higher in RBD as compared to KBD and WBD (*p* < 0.05), whereas fat intake tended to be opposite to carbohydrate intake in order WSD, KBD, and RBD. Similar to energy intake, protein intake was lower in RBD and WSD than KBD. The fiber intake per 1000 calories was significantly higher in KBD than in WSD and RBD. The participants in KBD consumed higher dietary fiber, Ca, vitamin C (V-C), vitamin A (V-A), and carotene per 1000 kcal intake than those in WSD and RBD (*p* < 0.05; Table 2). However, Na intake was higher in the RBD than the other groups (*p* < 0.05; Table 2). A higher percentage of the participants in the WSD performed physical activity and smoked more than the KBD and RBD. The participants with the KBD had the least alcohol consumption among all groups (Table 2). These results suggested that the participants in the KBD had better nutrient intake and lifestyles than other groups.

### 3.3. Enterotypes in the Korean Population

**Figure 2:**
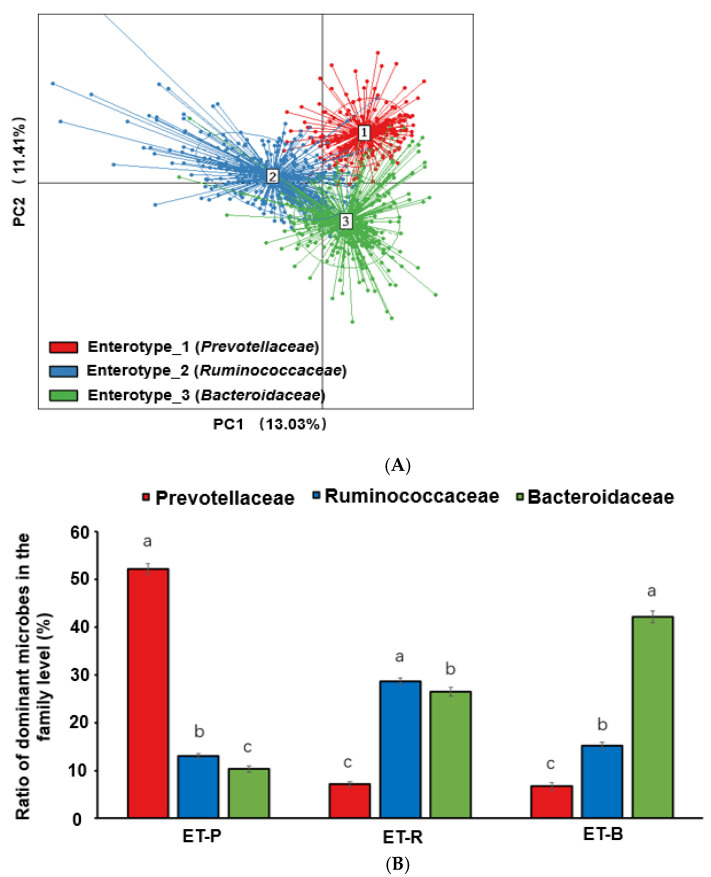
The classification of fecal bacteria and the dominant fecal bacteria in the family level according to each enterotype. (**A**) Enterotypes in the genus level by principal component analysis. (**B**) The relative proportions of dominant bacteria in the family level. Different alphabets (a,b,c) indicated significant differences among the different bacteria in the family level by Tukey’s test at *p* < 0.05.

Figure 2A showed that enterotype 1 was distinct from enterotype 2 and enterotype 3, while enterotypes 2 and 3 had some overlap. PC1 and PC2 had 13.03, and 11.41% explained variance, respectively. At the family level, the dominant bacteria of enterotype 1 was *Prevotellaceae* (ET-P), which was significantly higher than the other two enterotypes (Figure 2B). The dominant bacteria of enterotype 2 and enterotype 3 were *Ruminococcaceae* (ET-R) and *Bacteroidaceae* (ET-B), respectively (Figure 2B) (*p* < 0.05). According to the NGS data of 1199 Koreans, there were 208 (17.4%), 344 (28.7%), and 647 (54.0%) individuals in ET-P, ET-R, and ET-B, respectively.

### 3.4. Differences in the Abundant Bacteria at the Order, Family, Genus, and Species Levels and α-Diversity among the Enterotypes

**Figure 3:**
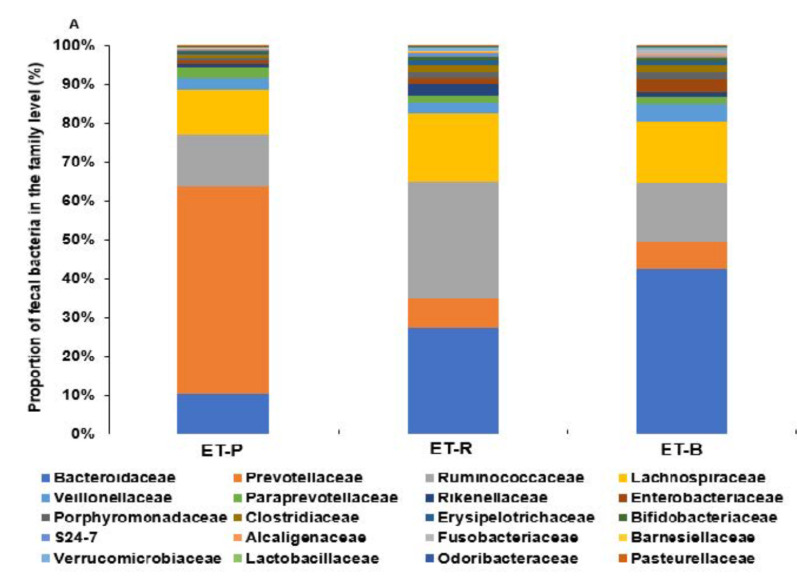

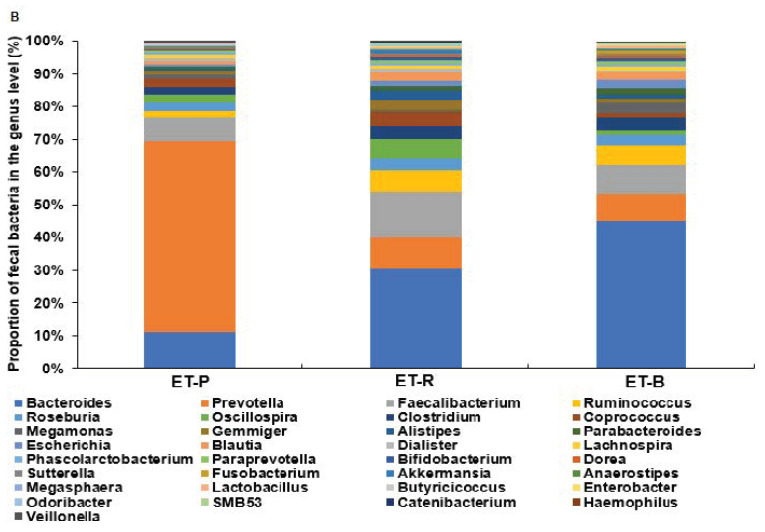

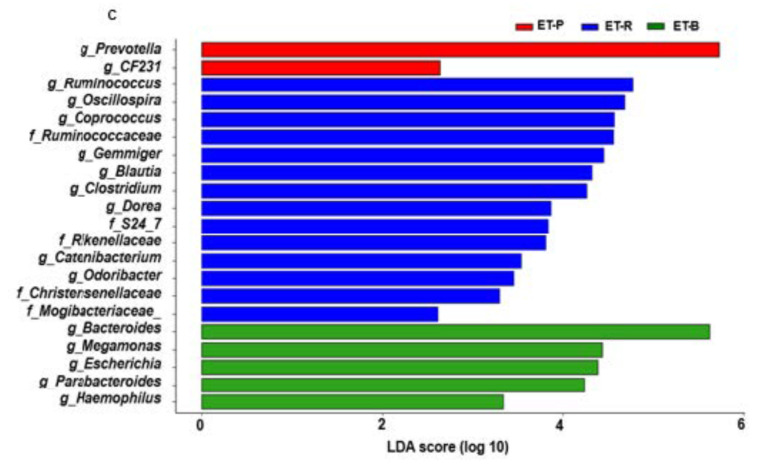
The relative abundance of the fecal bacteria in each enterotype at the (**A**,**B**) family level and genus level. (**C**) Primary bacteria in genus level by operational taxonomic unit (OTU)-based linear discriminant analysis effect size (LEfSe) analysis with the logarithmic discriminant analysis (LDA) score threshold >2.0 that was statistically significant at *p* < 0.05. ET-P, *Prevotellaceae*; ET-R, *Ruminococcaceae*; ET-B, *Bacteroidaceae*.

Fecal bacteria were classified into enterotype 1, enterotype 2, and enterotype 3 in the family level (Figure 3A) and genus levels (Figure 3B). At the family level, *Prevotellaceae* in the ET-P was significantly higher than that of other bacteria. The dominant bacteria of ET-R and ET-B were *Ruminococcaceae* and *Bacteroidaceae,* respectively (Figure 3A). However, ET-R also had a higher proportion of *Bacteroidaceae* than ET-P (*p* < 0.05; Figure 3B). At the genus level, ET-P and ET-B predominantly contained *Prevotella* (57.4%) and *Bacteroides* (45.3%), whereas ET-R rather than the presence of dominant bacteria (Figure 3B).

In LEfSe analysis, the primary bacteria in each dietary pattern were identified in genus level by the LDA >2.0 (*p* < 0.05), and unidentified bacteria in genus level were indicated as a family name (Figure 3C). ET-P mainly included *Prevotella*. ER-R highly contained *Ruminococcus*, *Oscillospira*, *Gemmiger*, and *Ruminococcaceae*, which belonged to *Ruminococcaceae*, and ET-R also included *Blautia* and *Coprococcus*, a family of *Lachnospiraceae* (Figure 3C). ET-R was mainly composed of *Ruminococcaceae* and *Lachnospiraceae*. ET-B included Bacteroides, Parabacteroides, and *proteobacteria (Escherichia,* and *Haemophilus)* (*p* < 0.05; Figure 3C).

The ET-R and ET-B contained significantly higher numbers of *Bifidobacteriales* than ET-P at the order level (Table 3). At the species level, ET-R had a significantly higher proportion of *Akkermansia muciniphila* compared to ET-P and ET-B. ET-R had a significantly higher proportion of *Bifidobacterium adolescentis* compared to ET-P and ET-B (Table 3). ET-R also had a significantly higher proportion of *Faecalibacterium prausnitzii* than ET-B, which was significantly lower in ET-P (*p* < 0.05). ET-P had the highest numbers of *Prevotella copri* compared to other enterotypes (*p* < 0.05) (Table 3).

**Figure 4:**
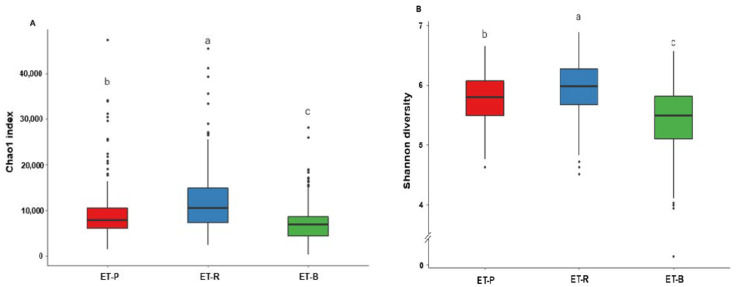
Alpha diversity in different enterotypes. (**A**) Chao1 index. (**B**) Shannon diversity. Different alphabets (a,b,c) represented significant differences among the different bacteria in the family level by Tukey’s test at *p* < 0.05. ET-P, *Prevotellaceae*; ET-R, *Ruminococcaceae*; ET-B, *Bacteroidaceae*.

ET-R exhibited the highest species richness (Chao1) and species evenness (Shannon), while ET-B showed the lowest number of species among all enterotypes (Figure 4A,B; *p* < 0.05).

### 3.5. Correlation of Foods in the Dietary Patterns and Fecal Bacteria

**Figure 5:**
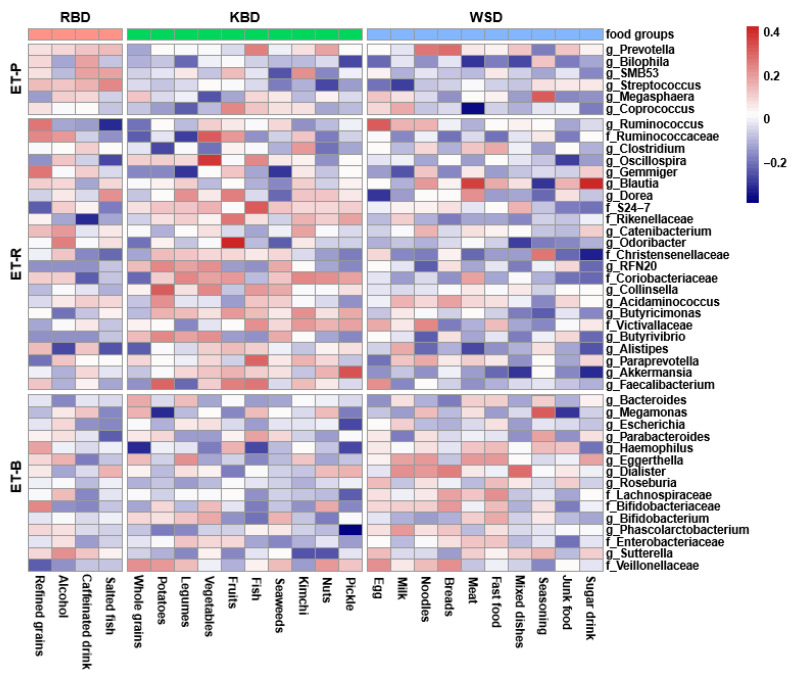
Heatmaps of partial Spearman correlations between intakes of food groups in each dietary pattern and relative abundance of gut microbiota at the genus level. Food groups were gathered into each dietary pattern, and fecal bacteria were arranged into one enterotype when the bacteria contents were higher than the other enterotypes, and they were classified into ET-P, *Prevotellaceae*; ET-R, *Ruminococcaceae*; ET-B, *Bacteroidaceae*.

Figure 5 presents the correlation matrix of food groups and fecal bacteria analyzed by partial Spearman correlations using CODA results. Food groups were gathered into each dietary pattern that was calculated by proc factor in Figure 5. Fecal bacteria were arranged into one enterotype when the bacteria contents were higher than the other enterotypes, and they were classified into ET-P, ET-R, and ET-B. The correlation matrix did not show a clear separation between enterotypes and dietary patterns. There were significant correlations between some food groups and specific fecal bacteria (*p* < 0.05) as follows: *Prevotella* has a positive correlation with breads (*r* = 0.29), noodles (*r* = 0.27), fish (*r* = 0.25), nuts (*r* = 0.18), salted fish (*r* = 0.14), mixed carbohydrate dishes (*r* = 0.14), and refined grains (*r* = 0.12). *Ruminococcus* has a positive correlation with egg (*r* = 0.33), fruits (*r* = 0.21), refined grains (*r* = 0.27), milk (*r* = 0.15), noodles (*r* = 0.148) and vegetables (*r* = 0.11). *Bacteroides* has a positive correlation with bread (*r* = 0.17), legumes (*r* = 0.14), seasonings (*r* = 0.12), fast food (*r* = 0.1) and meat (*r* = 0.11) (Figure 5). It showed the trend that the participants with RBD had higher in ET-P, those with KBD contained the relatively higher in ET-R, and those with WSD included relatively higher in ET-B and relatively lower in ET-R. These results indicated that the participants consuming high carbohydrate foods like refined grains, vegetables, noodles, and bread had a higher *Prevotella*. The participants having high fermented foods and vegetables had higher in ET-R, including *Akkermentia*.

### 3.6. Metabolic Activities of the Intestinal Bacteria in Each Enterotype by PICRUSt2

**Figure 6:**
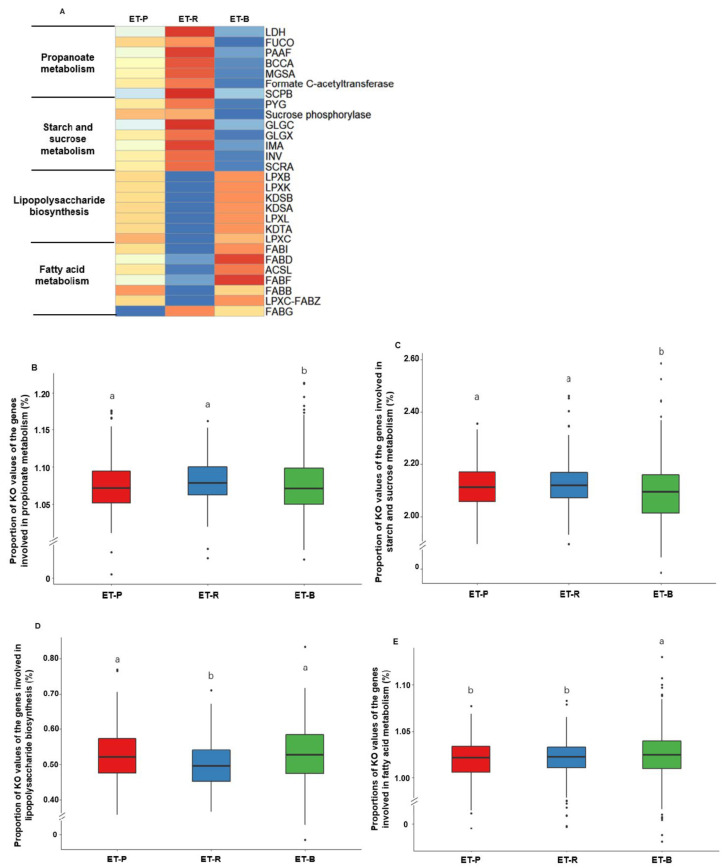
Heatmaps of abundant genes involved in metabolic functions determined by (**A**) PICRUSt2 and (**B**–**E**) the relative abundance of the genes related to significant metabolic functions in three enterotypes. (**A**) The association of the genes related to different functions in three enterotypes. (**B**) Propionate metabolism in three enterotypes. (**C**) Starch and sucrose metabolism in three enterotypes. (**D**) Lipopolysaccharide biosynthesis in three enterotypes. (**E**) Fatty acid metabolism in three enterotypes. LDH, L-lactate dehydrogenase; Figure 1. phosphate adenylyltransferase; GLGX, glycogen debranching enzyme; IMA, oligo-1,6-glucosidase; INV, beta-fructofuranosidase; SCRA, sucrose phosphoenolpyruvate-dependent sucrose phosphotransferase system permease component; LPXB, lipid-A-disaccharide synthase; LPXK, tetraacyldisaccharide 4′-kinase; KDSB, 3-deoxy-mannooctulosonate cytidylyltransferase; KDSA, 2-dehydro-3-deoxyphosphooctonate aldolase; LPXL, Kdo2-lipid IVA lauroyltransferase/acyltransferase; KDTA, 3-deoxy-D-manno-octulosonic-acid transferase; LPXC, UDP-3-O-(3-hydroxymyristoyl) N-acetylglucosamine deacetylase; FABI, enoyl-[acyl-carrier protein] reductase I; FABD, [acyl-carrier-protein] S-malonyltransferase; ACSL, long-chain acyl-CoA synthetase; FABF, 3-oxoacyl-[acyl-carrier-protein] synthase II; FABB, 3-oxoacyl-[acyl-carrier-protein] synthase I; LPXC-FABZ_2, UDP-3-O-[3-hydroxymyristoyl] N-acetylglucosamine deacetylase/3-hydroxyacyl-[acyl-carrier-protein] dehydratase; FABG, 3-oxoacyl-[acyl-carrier protein] reductase. KO, KEGG Orthologues. Different alphabets (a,b,c) represented significant differences among the different bacteria in the family level by Tukey’s test at *p* < 0.05. ET-P, *Prevotellaceae*; ET-R, *Ruminococcaceae*; ET-B, *Bacteroidaceae*.

The abundance of KEGG Orthologues involved in propanoate metabolism, starch and sucrose metabolism, LPS biosynthesis, and fatty acid metabolism are presented in Figure 6A, respectively (*p* < 0.05). The relative abundance of KEGG Orthologues involved in the propionate metabolism was significantly higher in ET-P and ET-R than ET-B (Figure 6B). ET-P and ET-R exhibited higher starch and sucrose metabolism than ET-B (*p* < 0.05; Figure 6C). The relative abundance of KEGG Orthologues involved in LPS biosynthesis was significantly higher in ET-P and ET-B than ET-R (*p* < 0.05; Figure 6D). Fatty acid metabolism was the highest in ET-B among all enterotypes (Figure 6E). Propionate metabolism and starch and sucrose metabolism were lower in ET-B than the other enterotypes (Figure 6B,C). LPS biosynthesis was lower in ET-R than in the other enterotypes, and fatty acid metabolism was higher in ET-B than in the other enterotypes (Figure 6D,E).

## 4. Discussion

In Korea, dietary patterns are primarily categorized into KBD, WSD, and RBD in KNHANES and Korean Genome and. Epidemiology Study (KoGES) cohorts [23,24]. KBD is similar to the other prudent diet patterns using KNHANES [23,25]. KBD contains a high total antioxidant capacity, and the persons having KBD has an inverse association with hypertriglyceridemia from KoGES and KNHANES [23]. RBD is associated with increasing metabolic syndrome risk in Asian countries, whereas KBD is related to lowering the risk [26,27]. The diets are associated with the gut microbiome that may influence metabolic syndrome risk [28]. We aimed to determine the association between metabolic syndrome and dietary patterns and determine the relationship between gut microbiota distribution and enterotypes in Korean adults. The potential connection between the dietary patterns and the enterotypes was also explored. The previous studies’ results of dietary patterns and metabolic syndrome in the present study were consistent [23,24,25,26,27]. Gut microbiota has been reported to a connection to the host’s metabolism. Dietary patterns influence gut microbiota, and enterotypes may be matched with the dietary patterns. Enterotypes have a significant correlation with a long-term diet [29]. The present study demonstrated that KBD had lower metabolic syndrome (MetS) and serum CRP concentrations, an inflammation index. Moreover, ET-R had a lower inflammatory response in fecal microbiota gene function analysis, and it showed a potential to have an association with KBD.

The enterotype concept was first introduced in 2011, and it was a more effective way to represent the diversity of intestinal microbes [8]. This classification of the intestinal microbial population has been reported to be affected by regional differences, including local dietary habits and indigenous bacteria [30,31,32], not by race, gender, or BMI [8]. According to a recent report, enterotypes have been mainly defined as *Prevotella*, *Bacteroidaceae*, and *Ruminococcaceae* types worldwide [33]. Although the enterotype names are used as *Prevotellaceae*, *Bacteroidaceae*, and *Ruminococcaceae,* each enterotype includes mixed bacteria. *Prevotellaceae* is included in *Bacteridetes* in the phylum level, and ET-P also contained highly *Bacteriodaletes* [34]. ET-B included mainly *Bacteroides*. However, the functional gene analysis shows ET-P Bacteria in ET-B are mainly associated with fat metabolism [35]. In the initial and subsequent studies of enterotypes, this classification was conducted mainly at the family and genus level [8,9]. To our knowledge, no study has reported the distribution of enterotypes in Korea compared to dietary types. Consistent with previous studies [36,37], Koreans were classified into three enterotypes—ET-P, ET-R, and ET-B—but ET-B and ET-R were not separated into two independent clusters and were found to be overlapping in the present study. Previous studies have also proposed that ET-R and ET-B are not discrete clusters in the classification but continuum [36].

To explore the correlation between Korea’s enterotypes and diet patterns, we conducted fecal microbiota analysis, the functionality of gut microbiota metabolism, and cluster analysis of dietary patterns in Korean adults in the present study. In PICRUSt2 analysis, ET-P was quite clearly separated from other enterotypes, and it had a high percentage of *Prevotellaceae* at the family level and *Prevotella* at the genus level. Similar to the previously reported results [38], ET-P showed significantly higher values for *Prevotella copri* than other enterotypes at the species level. ET-P significantly lowered for *Akkermansia mucinphila*, *Bifidobacterium adolescentis*, and *Faecalibterium prausnitzii* than ET-R. ET-P had no outstanding outcome in gut microbial function from PICRUSt2 analysis, but there were some significant differences in propionate metabolism, starch, sucrose metabolism, and LPS production in the present study. Advance research has reported that excessive *Prevotella copri* could over-activate toll-like receptor 4, up-regulate inflammatory pathways such as c-Jun N-terminal kinase and nuclear factor-κB to increased rheumatoid arthritis risk [39,40,41,42]. The low concentrations of *Faecalibterium prausnitzii* and *Akkermansia muciniphila* could lead to an increase in LPS production [43]. Due to these results, the ET-P type, which was predominated by *Prevotella copri* and had less *Akkermansia muciniphila* and *Faecalibterium prausnitzii*, showed high starch and sucrose metabolism and LPS metabolism [43]. These results are consistent with our results, which showed high LPS production in ET-P with the abundance of *Prevotella* [36,37], and RBD had higher refined grain, alcohol, and caffeinated drink intake than others. According to microbe function (starch and sucrose metabolism, and LPS production), nutrient intake (high intake of refined grain, alcohol, and caffeinated drink), and the anthropometric and biochemical results (relatively high level of hemoglobin A1c, serum triglyceride, and WBC), we estimated ET-P as RBD in the present study. Following research supported our results, indicating that people with ET-P have a long-term intake of a diet rich in carbohydrates and simple sugars [13]. ET-P and RBD showed similar results in the Korean population.

*Ruminococcaceae* at the family level and *Alistipes* at the genus level showed higher than others in a proportion of bacteria. At the species level, *Akkermansia muciniphila* was highest in ET-R, and *Bifidobacterium adolescentis* and *Faecalibterium prausnitzii* were also abundant in this enterotype in the present study. Although not yet identified about *Alistipes*, it has been interpreted as a healthy phenotype in cardiovascular diseases and inflammatory diseases such as colitis and hepatitis, and pathogenic and protective effects coexist in mental diseases [44]. *Akkermansia muciniphila* adheres to the host’s intestinal mucus layer and degrades the mucin layers to obtain energy and carbon sources, which in turn, it releases physiologically active substances such as SCFAs that change the composition of mucus [45]. Mucin contents influence the host’s signal transduction pathway to maintain intestinal morphological integrity [46]. It is known that *Bifidobacterium adolescentis* have anti-obesity and anti-inflammatory activities [47], and *Faecalibterium prausnitzii* is an excellent butyric acid-producer [42]. Butyrate can stimulate colon mucin and enhance the barrier capacity of the intestine [41]. In addition, the intake of *Faecalibterium prausnitzii* can promote the production of butyric acid and increase the abundance of *Akkermansia muciniphila*, thereby ensuring the integrity of the intestinal barrier in vivo [48]. Because ET-R has many SCFA-producing bacteria with low LPS-producing ability, the anti-inflammatory response might be the highest among the three enterotypes. In the dietary patterns, KBD had a higher intake of potatoes, legumes, vegetables, fish, seaweeds, kimchi, and pickles, and they had a higher intake of energy, protein, total fiber, and V-C. The persons having the KBD were lower in the blood concentrations of hemoglobin A1c, triglyceride, CRP, and WBC than those having other diets in the present study. Consistent with our study, those with a KBD have shown high anti-inflammatory properties and the lowest incidence of metabolic syndrome in previous studies [49,50,51]. These results suggest that the adults with KBD had a lower inflammatory response than those with RBD or WBD [49]. Therefore, ET-R was interpreted as KBD, which has a typical anti-inflammatory function.

Finally, despite incompletely separated from ET-R, ET-B contained prevalent *Bacteroidaceae* in the family and *Bilophila* in the genus level. Previous studies have shown that *Bacteroides* enterotype has significant interaction with the Western-style diet [52], and persons with ET-B have consumed high animal protein and animal fat diets for long periods [29,53]. *Bilophila* tends to increase with the animal-based diet, which indicates *Bilophila* has a linkage with dietary fat intake [54]. ET-B showed high fatty acid metabolism and LPS production (Figure 4), a common endotoxin in the human body. LPS produced by intestinal bacteria can trigger systemic and local pro-inflammatory and immunomodulatory responses [55]. In the research of the intestinal type of diabetic patients, it also has concluded that ET-B has more LPS and TNF-α content [56]. In dietary pattern and nutrient intake, WSD showed a high intake of noodles, bread, fast foods, and sugar drink, which contained a high-fat percentage. In enterotype’s microbial distribution in the genus level, the proportion of *Bilophila,* bacteria having an interaction with animal-protein intake in ET-B, was relatively higher than ET-R, whereas, at the species level, the proportion of *Akkermentia mucinphila* and *Faecalibterium prausnitzii* in ET-B was also lower than ET-R in the present study. In WSD, fat intake and serum CPR, a systemic inflammation marker [39], were higher than KBD. The relative abundance of *Akkermentia muciniphila* was lower, which induced the weakening of the intestinal mucin barrier’s barrier capability. It might link to cause systemic inflammation as the LPS produced in the intestine moves from the intestine to the blood and circulates.

This study has several limitations. Because the study was conducted using a case-control design, its results cannot establish cause-and-effect relationships, although this study had the advantage of a large sample size. The results between the enterotypes and dietary patterns explained potential possibilities. It is necessary to clarify the relationship between gut microbes and the Korean dietary types through targeted research in the future based on NGS and dietary intake of the same population.

## 5. Conclusions

Based on the nutrition and health data obtained by KNHANES, the Koreans were classified into three categories according to dietary patterns, namely WSD, KBD, and RBD. Adults with RBD had a positive association, and those with KBD exhibited a negative relation with metabolic syndrome in KNHANES. Korean adults showed the presence of three enterotypes, including ET-P, ET-R, and ET-B. There were potential connections between enterotypes and dietary patterns. The enterotype ET-P was associated with higher starch and sucrose metabolism and SCFA producing capacity, seen in those on a high carbohydrate diet (RBD) and consuming bread and noodles. ET-R had a lower inflammatory response, which was possibly linked to the adults with a KBD. ET-B was shown to have the potential to be related to those on a WSD with high-fat and protein diets but a relatively high dietary fiber intake. Therefore, the food components in the WSD in Koreans were different from the Western countries, and ET-B in Koreans might be different from the Caucasians. The interactions of gut microbiota and dietary patterns with metabolic syndrome may predict individual metabolic disorders from the dietary pattern or enterotype using a machine learning approach. Thus, the relationship between dietary patterns and fecal bacteria needed to be further elucidated.

## Figures and Tables

**Table 1:** Anthropometry and biochemical measurements in three dietary patterns.

	Rice-Based Diet (*n* = 1542)	Korean Balanced Diet (*n* = 1825)	Western-Style Diet (*n* = 5577)
Age (years)	44.6 ± 0.43 ^a^	43.7 ± 0.37 ^a^	39.1 ± 0.20 ^b^
Gender (male *N*, %)	1148(74.5)	569(31.2)	2065 (37.0) ***
Metabolic syndrome (*N*, %)	219 (14.2)	136 (7.43)	586 (10.5) ***
Body mass index (kg/m^2^)	23.5 ± 0.13	23.3 ± 0.11	23.4 ± 0.07
Waist circumferences (cm)	80.0 ± 0.34	79.5 ± 0.28	79.8 ± 0.15
Serum glucose (mg/dL)	95.9 ± 0.62	94.0 ± 0.53	94.9 ± 0.28
Hemoglobin A1c (%)	5.52 ± 0.02 ^a^	5.47 ± 0.02 ^b^	5.47 ± 0.01 ^b^
Serum total cholesterol (mg/dL)	192 ± 1.25	191 ± 1.06	192 ± 0.57
Serum HDL (mg/dL)	52.2 ± 0.45	53.0 ± 0.39	53.1 ± 0.21
Serum LDL (mg/dL)	119 ± 1.69	116 ± 1.95	116 ± 1.11
Serum triglyceride (mg/dL)	135 ± 3.9 ^a^	120 ± 3.4 ^b^	126 ± 1.8 ^a,b^
Serum CRP (mg/mL)	1.10 ± 0.09 ^a^	0.97 ± 0.07 ^b^	1.12 ± 0.04 ^a^
Serum AST (IU/L)	20.9 ± 0.46	21.3 ± 0.39	21.2 ± 0.21
Serum ALT (IU/L)	21.3 ± 0.71	21.4 ± 0.60	20.9 ± 0.32
Systolic blood pressure (mmHg)	112 ± 0.48	113 ± 0.41	113 ± 0.22
Diastolic blood pressure (mmHg)	75.0 ± 0.34	74.6 ± 0.29	74.8 ± 0.15
Serum folate (ng/mL)	6.37 ± 0.26 ^b^	7.32 ± 0.23 ^a^	6.92 ± 0.11 ^a,b^
Serum V-A (mg/L)	0.49 ± 0.02	0.44 ± 0.01	0.44 ± 0.01
Serum 25-hydroxy cholecalciferol (ng/mL)	15.3 ± 0.55	15.3 ± 0.54	15.9 ± 0.34
Blood platelet (×10^9^/L)	271 ± 2.53 ^a^	259 ± 2.21 ^b^	262 ± 1.16 ^b^
White blood cell counts (×10^3^/µL)	6.73 ± 0.06 ^a^	6.21 ± 0.05 ^b^	6.27 ± 0.03 ^b^

Values represented means and standard deviations. Adjusted for age, sex, body mass index, smoking and drinking status, and physical activity. ^a,b^ Different letters represented significant differences at *p* < 0.05. *** Significant among the groups at *p* < 0.001. HDL, high-density lipoprotein; LDL, low-density lipoprotein; CRP, C-reactive protein; AST, aspartate aminotransferase; ALT, alanine aminotransferase; V-A, vitamin A.

**Table 2:** Nutrient intake calculated from semi-quantitative food frequency questionnaires in three dietary patterns.

	Rice-Based Diet (*n* = 1542)	Korean-Balanced Diet (*n* = 1825)	Western-Style Diet (*n* = 5577)
Energy (EER%)	104 ± 2.06 ^b^	116 ± 1.69 ^a^	106 ± 0.88 ^b^
Carbohydrate (energy%)	67.1 ± 0.73 ^a^	62.3 ± 0.56 ^b^	62.3 ± 0.30 ^b^
Fat (energy%)	19.2 ± 0.32 ^c^	22.1 ± 0.31 ^b^	23.9 ± 0.16 ^a^
Protein (energy%)	13.7 ± 0.17 ^b^	15.3 ± 0.16 ^a^	13.7 ± 0.09 ^b^
Total fiber (g/day)	24.5 ± 0.58 ^b^	28.7 ± 0.64 ^a^	23.8 ± 0.27 ^b^
Ca (mg/day)	521 ± 15 ^b^	592 ± 19 ^a^	501 ± 6.0 ^b^
Na (mg/day)	4445 ± 195 ^a^	4165 ± 79.7 ^b^	3823 ± 55.6 ^c^
V-C (mg/day)	89.4 ± 5.2 ^b^	120 ± 5.3 ^a^	88.5 ± 2.4 ^b^
V-A (RE/day)	712 ± 39 ^b^	856 ± 54 ^a^	702 ± 20 ^b^
Carotene (mg/day)	3483 ± 171 ^b^	4071 ± 316 ^a^	3224 ± 106 ^b^
Physical activity (yes *N*, %)	631 (47.6)	975 (58.8)	2648 (52.5) ***
Drinking (yes *N*, %)	640(48.3)	452 (27.3)	1313 (26.0) ***
Smoking (yes *N*, %)	358 (27.0)	1177 (71.0)	3408 (67.6) ***

Values represented adjusted means and standard deviations after adjusting for age, sex, body mass index, smoking and drinking status, and physical activity. ^a,b,c^ Different letters represented significant differences at *p* < 0.05. *** Significant among the groups at *p* < 0.001.

**Table 3:** Gut microbiomes in order and species levels (unit: %).

Bacteria	Level	*Prevotellace*	*Ruminococcaceae*	*Bacteroidaceae*
*Bifidobacteriales*	Order	0.26 ± 0.04 ^b^	0.87 ± 0.09 ^a^	0.92 ± 0.14 ^a^
*Lactobacillales*	Order	0.35 ± 0.06	0.49 ± 0.09	0.67 ± 0.20
*Alistipes*	Genus	0.57 ± 0.07 ^c^	2.25 ± 0.15 ^a^	1.16 ± 0.11 ^b^
*Bilophila*	Genus	0.01 ± 0.002 ^b^	0.02 ± 0.002 ^b^	0.04 ± 0.01 ^a^
*Akkermansia muciniphila*	Species	0.05 ± 0.02 ^b^	0.60 ± 0.12 ^a^	0.28 ± 0.09 ^b^
*Bifidobacterium adolescentis*	Species	0.18 ± 0.03 ^b^	0.49 ± 0.07 ^a^	0.31 ± 0.08 ^ab^
*Faecalibacterium prausnitzii*	Species	6.71 ± 0.35 ^c^	11.86 ± 0.46 ^a^	8.19 ± 0.42 ^b^
*Prevotella copri*	Species	50.5 ± 1.05 ^a^	5.94 ± 0.44 ^b^	5.49 ± 0.76 ^b^

Values represented means and standard deviations. ^a,b,c^ Different alphabets represented significant differences among the different bacteria in the family level by Tukey’s test at *p* < 0.05.

## Data Availability

The data are available from the corresponding author on reasonable request.
